# Hypertension: a problem of organ blood flow supply–demand mismatch

**DOI:** 10.2217/fca.16.5

**Published:** 2016-04-19

**Authors:** Maarten P Koeners, Kirsty E Lewis, Anthony P Ford, Julian FR Paton

**Affiliations:** 1 School of Physiology, Pharmacology & Neuroscience, Biomedical Sciences, University of Bristol, Bristol, UK; 2 Afferent Pharmaceuticals, 2929 Campus Drive, San Mateo, CA, USA

**Keywords:** hypertension, hypoperfusion, organ blood flow, sympathetic hyperactivity, visceral afferent hyper-reflexia

## Abstract

This review introduces a new hypothesis that sympathetically mediated hypertensive diseases are caused, in the most part, by the activation of visceral afferent systems that are connected to neural circuits generating sympathetic activity. We consider how organ hypoperfusion and blood flow supply–demand mismatch might lead to both sensory hyper-reflexia and aberrant afferent tonicity. We discuss how this may drive sympatho-excitatory-positive feedback and extend across multiple organs initiating, or at least amplifying, sympathetic hyperactivity. The latter, in turn, compounds the challenge to sufficient organ blood flow through heightened vasoconstriction that both maintains and exacerbates hypertension.

**Figure 1.**
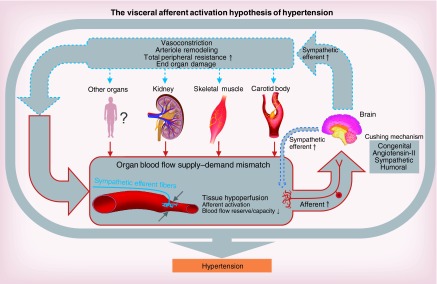
**Visceral afferent activation hypothesis of hypertension.** When afferents become sensitized due to, for example, high sympathetic activity, circulating angiotensin II, atheroma or congenital predisposition (although exact mediator[s] remain to be identified) they reflexly increase sympathetic vasomotor tone reducing making an organ vulnerable to hypoxic-hypoperfusion and reducing its blood flow reserve/capacity. We propose that this triggers release of metabolites that activate excessive afferent nerve activity, which exacerbate the reflex-evoked sympathetic vasoconstriction. Widespread sympathetic activation may recruit additional afferent systems resulting in additional drivers for maintaining pathologically high levels of sympathetic activity and total peripheral resistance. Thus, the system degenerates into a positive feedback loop in hypoxic-hypoperfusion and reduced blood flow reserve/capacity acts as an amplifier and conceivable initiator of sympatho-excitatory afferent drive. Consequently, vasoconstriction, arteriole remodeling increased total peripheral resistance, and end organ damage will amplify the organ blood flow supply–demand mismatch compounding the problem of hypoperfusion and the development and maintenance of hypertension. We do not rule out that excessive sympathetic activity itself sensitizes the afferent nerves mediating the reflex-evoked sympatho-excitation. Artwork was provided by Michel Cekalovic [12].

## Background

Causes of elevated sympathetic activity in neurogenic hypertension remain enigmatic. We explore evidence to support the notion of blood flow supply–demand mismatch to an organ as a factor involved in the initiation of raised sympathetic activity, and its subsequent amplification, in hypertension. We first consider some homeostatic mechanisms regulating blood flow and then discuss emerging changes that may occur during the development of hypertension that sensitize afferent innervation of target organs. We hypothesize that activation of visceral afferent systems induces increases in sympathetic nerve activity (SNA) that can initiate and sustain hypertension. We term this the visceral afferent activation hypothesis of hypertension.

### • Physiological mechanisms ensuring organ blood flow homeostasis: a brief overview

All organs have a capacity to increase blood flow through local functional (reactive) hyperemia, autoregulatory, endocrine and neural mechanisms. Blood flow autoregulation is defined as the ability of an organ to maintain a constant blood flow despite either increases or decreases in blood (perfusion) pressure. Blood flow autoregulation is intrinsic to the arterioles within the organ and occurs in the absence of neural and hormonal influences. Endocrine controls include epinephrine and norepinephrine, as well as vasopressin, the renin–angiotensin–aldosterone system, atrial natriuretic peptide and erythropoietin. Neural mechanisms include baroreceptors in the aorta, carotid sinuses and right atrium, and peripheral and central chemoreceptors that monitor blood levels of oxygen, carbon dioxide and hydrogen ions; together these systems modulate levels of autonomic and hormonal activity through central reflex arcs coursing through various interconnected CNS structures. Such structures also bring about blood flow homeostasis during stress, hyperosmolarity, emotion, fear and anxiety. In physiological conditions, the aforementioned homeostatic mechanisms will distribute blood flow, and hence oxygen, to organs in proportion to their metabolic demand, which is precisely controlled [1]. Normally, oxygen delivery exceeds the rate of oxygen consumption (demand) safeguarding capacity within each organ, which can be tapped into as a change in state demands [1]. For example, upon ascending to high altitude or during exercise, blood flow is increased to maintain a preferred supply/demand ratio [1]. However, if demand outstrips supply (i.e., a blood flow supply–demand mismatch), then hypoxic-hypoperfusion results. This might be corrected by increasing perfusion pressure, perhaps evoked through excitation of the organ's own afferent system. However, according to Poiseuille's Law, at constant perfusion pressure blood flow is directly proportional to the fourth power of the radius of the vessel, meaning that a proportionally large increase in blood pressure will be needed to restore blood flow if the radius decreases, that is, the resistance increases. Blood pressure can be elevated by increasing either cardiac output and/or vascular resistance through activation of a neurohumoral mechanism including vasomotor sympathetic activity. Once the organ's oxygen demand returns to basal levels, hemodynamic homeostasis is restored. However, if organ hypoxic hypoperfusion persists, or if the afferent circuits from that organ convey signals of such condition, even if aberrantly once it has passed, we surmise that this may result in augmented sympathetic discharge and, thus, hypertension. It has been maintained that most forms of hypertension are associated with increased total peripheral resistance (TPR), rather than increased cardiac output [2]. In such conditions, increased SNA in both men and older women is a primary driver of increased TPR [3]. Chronic elevation of TPR as a mechanism to increase blood pressure may reduce organ blood flow capacity, thereby increasing the vulnerability to tissue hypoxic-hypoperfusion. Since all organs are innervated by sensory nerves, we propose that organ hypoperfusion will activate afferent activity and reflexly stimulate sympathetic hyperactivity leading to hypertension. However, this generalized vasoconstriction may compromise blood flow to other organs and thus trigger blood flow a broad supply–demand mismatch, which may result in the recruitment of additional afferent drivers that compound the hypertensive state. We explore this cycle of maladaptation in more detail giving examples to substantiate this notion.

## The sympathetic problem of hypertension

Unequivocal evidence supports chronic activation of the sympathetic nervous system as a characteristic of hypertension and its participation in the initiation, maintenance and progression of blood pressure in experimental animal and human studies [4]. A big clinical challenge is to unveil the cause(s) of sympathetic overactivation and design novel therapies that lower it below its threshold for triggering significant increases in TPR. Evidently, sympathetic hyperactivity interacts with many pathologies beyond neurogenic hypertension, including heart failure, salt sensitivity, obesity, glucose intolerance, rheumatoid arthritis and many more. However, the aim of our review is to understand the relationship between organ hypoperfusion and subsequent visceral afferent activation as a potential driver of sympathetically mediated hypertension.

## Visceral afferent hyperactivity: consequences for sympathetic activity & organ blood flow

To the best of our knowledge, all organs are connected to the brain reciprocally by visceral afferent and efferent nerves (Figure 1). Many of these afferent nerves can trigger sympatho-excitatory responses through spinal and supra-spinal reflexes. Exceptions include baroreceptors, right atrial and pulmonary stretch receptors and some renal afferents that inhibit sympathetic activity and may oppose hypertension-induced sympathetic hyperactivity [5]. Interestingly, these receptors are mechanosensory and deactivated by hypoperfusion and/or metabolic stimuli so may not be relevant to blood flow supply–demand mismatch [5]. We propose that metabolite-sensitive afferents (metabosensors) that trigger sympathetic activity responses are less susceptible to desensitization (adaptation) and, under pathophysiological conditions can: become sensitized, producing exaggerated reflex responses (hyper-reflexia); and generate aberrant tone (hypertonicity); the latter has the potential to provide a sustained drive for maintaining high levels of sympathetic activity (Figure 1). Their activation may actively depress sympatho-inhibitory reflexes, such as the arterial baroreceptor reflex. Evidence supporting the presence of excessive afferent signaling in hypertension comes from the kidney [6,7] and carotid body [8–11] as their selective denervation can reduce both sympathetic activity and blood pressure, as we discuss later.

It is important to recognize that physiological afferent signaling from an organ may occur in response to stimulants released locally because of a change in metabolic demand. Such examples include: reduced blood flow and hypoxia, hypercapnia and metabolic stress. Functional or reactive hyperemia in skeletal muscle is a mechanism that equates the change in metabolic demand with increases in blood flow. The metabolites leached from respiring tissues cause localized vasodilatation but, at sufficient concentration, also stimulate afferent endings (group III or IV fibers). The latter results in reflex increase in sympathetic activity and arterial pressure, with the sole aim of increasing perfusion to the muscle bed from which the afferents were activated. Functional hyperemia normally offsets sympathetically mediated vasoconstriction [13]. The net result is elevated blood flow to the metabolically active organ to satiate its increased demand for oxygen. The increased perfusion also serves to washout metabolites, reduce their accumulation and may temper afferent activity to a level that optimizes oxygen supply and demand; hence an optimal equilibrium is reached. Afferent signaling normally ceases once oxygen supply outstrips demand. However, in disease states, such as hypertension, afferent signaling may become exaggerated and persist even in the absence of metabolites. Further, local metabolites mediating the vasodilatation may be less effective at opposing high levels of sympathetically mediated vasoconstriction [14,15]. Indeed, in hypertension, nitric oxide did not attenuate sympathetic vasoconstriction during exercise – an effect seen in animals with normal blood pressure [14]. We discuss these possibilities below.

### • Sensitization of afferent feedback generating sympathetic excess in hypertension

Sympathetic activation patterns may be graded and/or differentially controlled on an organ basis. For example, the classic ‘defense’ or ‘alerting’ response is characterized by increases in arterial pressure and heart rate with vasodilatation in skeletal muscle (mediated, in part, by sympatho-inhibition), and vasoconstriction in the splanchnic, renal and cutaneous vascular beds [16]. Our notion is that in conditions of hypertension there is an underlying imbalance, with vasodilatory metabolites giving way to predominance of sympathetic vasoconstrictor influence that is driven by afferent sensitization and emergence of aberrant afferent tonicity. The mechanism(s) involved in afferent sensitization and tone generation remain an open question and may be organ and state dependent but could result as a consequence of hypoxic-hypoperfusion triggering transcriptional processes. We propose that a plethora of initiators/mediators could be involved in this sensitization, including metabolic stressors (hypoxia, hypercapnia, hyperglycemia, ATP, potassium ions, low pH), oxidative stress, anatomical anomalies (preconditioned, congenital), anemia, respiratory stress, atherosclerotic plaque, inflammation and renin–angiotensin system (RAS) activation. These mediator(s) themselves may sensitize the very mechanisms that cause depolarization and trigger receptor potentials in afferent endings resulting in a chronic excitatory effect. Important to our hypothesis is that aberrant afferent firing persists at rest in the absence of any metabolic stimuli and, thus, is a pathological signal. The latter is highly relevant as at rest there will be an absence of opposing vasodilatory metabolites making sympathetically mediated vasoconstriction more intense, worsening the problems of hypoperfusion and hypertension. Through a vicious circle, the latter would result in further stimulation of afferent activity and reflexly evoked sympathetic vasoconstriction accentuating the problem of blood flow supply–demand mismatch. We do not rule out a crosstalk mechanism, whereby sympathetic activity itself sensitizes adjacent afferent endings as an additional amplifying mechanism; there is at least anatomical evidence for this in the kidney where afferents and sympathetic fibers are intertwined [17]. Highly analogous is somatosensory sensitization seen in states of ‘sympathetically maintained pain’ or reflex sympathetic dystrophy, now thought of as ‘complex regional pain syndrome’. These are chronic pain conditions associated with sympathetic sprouting onto primary afferents terminals, dorsal root ganglion and spinal dorsal horn afferent terminals [18]. Being released by sympathetic postganglionic neurones, ATP participates in this sensitization [19]. We propose that the same process of sensitization may apply to visceral afferent activation of hypertension.

Heightened activation of the sympathetic nervous system through aberrant afferent tonicity from an organ may offset the vasodilatation produced by functional hyperemia. For example, abnormal muscle afferent signaling in heart failure patients increased the passive leg movement-induced increases in norepinephrine spillover and arterial blood pressure, which significantly reduced femoral blood flow, oxygen delivery and tissue oxygen saturation [20]. If sympathetically mediated vasoconstriction becomes widespread across vascular beds this may recruit multiple sympatho-excitatory afferent systems and amplify the problem of raised blood pressure (Figure 1). Additionally, autoregulation in response to the rising perfusion pressure will also increase vascular resistance in an attempt to maintain constant blood flow, but at the expense of further elevating TPR. Moreover, persistent and heightened sympathetic activity may reset autoregulation to higher pressure levels (e.g., renal blood flow autoregulation curve is shifted to a higher range of renal arterial pressures in hypertensive dogs with enhanced sympathetic activity [21]), as well as cause arteriolar remodeling [22]; this would have deleterious consequences for blood flow supply–demand matching. Finally, it is also likely that hypoxic-hypoperfusion and reduced blood flow become greatly agonized at night when blood pressure normally dips [23]. In a constant state of sympathetic overactivity, it is easy to see how nocturnal dipping is lost in conditions of hypertension [24]. Below we consider some examples of organs exhibiting excessive afferent activity and heightened SNA in hypertension.

## Potential sources of afferent hyperactivity

Although it seems that many, if not all, organs affect blood pressure regulation, we will focus within this review on the organs that we consider as exemplary of our hypothesis and where evidence exists supporting organ hypoperfusion and blood flow supply–demand mismatch triggering dysfunctional afferent activity and reflexly driven sympathetic activity.

### • Kidney afferents & hypertension

Renal afferent nerves primarily terminate peripherally in the renal pelvis and project to the spinal cord with onward transmission to the nucleus tractus solitarii, rostral ventrolateral medulla (RVLM), subfornical organ and paraventricular nucleus (PVN). There are single modality fibers (mechanoreceptors) that sense stretch of the pelvic wall causing sympatho-inhibition, chemosensitive receptors causing sympatho-activation and multimodal fibers that sense both (reviewed in [25]). Neurally mediated responses occurring in one kidney as a result of interventions on the same or opposite kidney have been defined as renorenal reflexes, which function to decrease renal efferent SNA to minimize sodium retention [26]. However, in ischemic/hypoxic kidneys and/or in the hypertensive state, there is a reversal of the renorenal reflex: stimulation of afferent nerves by ischemic metabolites, such as adenosine, and/or by uremic toxins, such as urea [27], which enhances the sympatho-excitatory state, increases salt retention and blood pressure. This reversal of the renorenal reflex is significant since afferent signals emanating from the kidney to the CNS have been strongly associated with the etiology of hypertension. Indeed in experimental animal models of hypertension, the removal of the afferents by either dorsal rhizotomy [7,28–30] or specific inhibition of afferent fibers by capsaicin treatment [6] have proven to be antihypertensive. Interestingly, Cowley *et al*. [31] has demonstrated that a shift in the redox balance and oxidative stress reduces the blood flow to the medulla, a mildly hypoxic area of the kidney, leading to hypertension and renal injury. This supports the concept of oxidative stress as an initiator/mediator involved in hypertension, potentially via hypoxia-hypoperfusion and afferent sensitization. Recent clinical trials have shown that blood pressure and SNA are both reduced after bilateral denervation (afferent and efferents) of the kidneys in some drug-resistant hypertensive patients [32–34], although the efficacy of this intervention is debated [35]. Recently, it has emerged that afferents are important for the hypertensive state in chronic kidney disease [36]. Future studies are needed to understand the mechanisms for renal afferent sensitization and tonicity in hypertensive patients; putative stimulants are discussed below. Hypothetically, understanding the latter may provide an effective means to select patients most likely to respond to treatment (pharmacological or denervation). Taken together, these data emphasize the role of afferent signals emanating from the kidney in the generation of hypertension.

In recent years, evidence has accumulated that kidney hypoxia plays a significant role in the pathogenesis and progression of renal disease [37–40]. Even a small lesion in the kidney, resulting in an area(s) of ischemia not necessarily affecting renal function, can cause hypertension, perhaps via afferent activation [41]. Renal hypoperfusion, ischemia and hypoxia can all trigger afferent signaling to reflexly elevate sympathetic activity and arterial pressure. This has led to the prediction that renal hypoxia is not just a consequence of kidney disease, but rather a primary pathogenic event [37–39,42–48]. But what could be the initial trigger? Exogenous administration of angiotensin II (AngII) can lead to hypoperfusion of postglomerular peritubular capillaries, and subsequent hypoxia within the tubules and interstitial space [40,49]. We propose that hypertensive kidney disease is driven by vicious loops of positive feedback, initiated by increased AngII triggered by hypoperfusion and accompanied by nephron loss, inflammation and diabetes. This results in oxidative stress and nitric oxide deficiency augmenting hypoperfusion and renal afferent signaling. Therefore, improving blood perfusion and thus oxygenation of the kidneys may reduce SNA and arterial pressure and improve renal function, thereby providing a novel future treatment strategy. This is supported by the findings that: pharmacologically increasing renal oxygen consumption *per se* caused renal hypoxia, proteinuria [50] and eventually leads to renal injury [51] and; application of 100% oxygen to patients with chronic kidney disease reduced muscle SNA, whereas such an intervention did not affect muscle SNA in control patients [52]. This mismatch in renal oxygen demand and supply exemplifies how afferent driven sympatho-excitatory is a powerful stimulus for hypertension.

### • Carotid body afferents & hypertension

Stimulation of the carotid body (CB), the dominant peripheral chemoreceptor, drives sympathetic tone through relatively direct signaling to the nucleus tractus solitarii, rostral ventrolateral medulla and paraventricular nucleus resulting in increased blood pressure [53,54]. In cardiovascular diseases, it appears that the CB generates aberrant afferent discharge [8,9]. Schultz *et al*. have clearly implicated a significant role of CB afferent drive in the sympathetic hyperactivity and breathing dysregulation in animal models of chronic heart failure [55,56]. Blood flow to the CB is exceptionally high in relation to tissue mass in health [57], but in rabbits with pacing-induced chronic heart failure this was reduced due to lowered cardiac output [56]. This might account for CB afferent tonicity. Given this, one might predict that lowering CB afferent drive should be therapeutically beneficial. As a proof of principle, CB resection was performed in heart failure patients and partially corrected cardiac autonomic balance and cardiac baroreflex gain [10,11] and reduced muscle sympathetic activity. Moreover, the CB is an afferent source driving sympathetically mediated hypertension in the spontaneously hypertensive rat (SHR) [8,9]. Again CB chemoreceptors are tonically active in the SHR [8,9] and their disconnection from the brain is antihypertensive, substantially reduces renal sympathetic activity, and improved both baroreflex and renal function [8,9]. Intriguingly, there was an additive blood-pressure lowering effect when renal denervation was subsequently performed [9]. These data strongly support our hypothesis that organ hypoperfusion and blood flow supply–demand mismatch recruits additional sources of afferent drive across organs with each independently contributing to sympathetic excess and hypertension (Figure 1).

### • Skeletal muscle afferents & hypertension

Sympathetic engagement during exercise is initiated, in part, by activation of thin-fiber afferents arising from contracting skeletal muscle. In the case of lower limb muscles, these project via the lumbar dorsal horn of the spinal cord and via supra-spinal circuits modulate cardiovascular and respiratory activity and their reflex control [58]. These afferents are mechanically (mechanoreceptor; type III sensory fibers) and/or metabolically sensitive (metaboreceptor; type IV fibers). During the onset of exercise, these afferents become activated and evoke the ‘exercise pressor response’ consisting of rises in arterial pressure, heart rate, stroke volume, cardiac output, redistribution of blood flow to active skeletal muscle and hyperventilation [59]. What evidence exists that these afferents become tonically active in the hypertensive state? In hypertension, sensitization of both mechanically and metabolically sensitive skeletal muscle afferents has been demonstrated [60,61]. Moreover, the exercise pressor response is augmented and associated with the development of hypertension [62]. In chronic heart failure, this includes an increased sensitivity of type 3 fibers compared with type 4 and heightened P2X response [63]. Functional sympatholysis, the process whereby sympathetic vasoconstriction is offset by metabolites released from the exercising skeletal muscle, is impaired substantially in hypertension [64]; this results in reduced oxygenation and blood flow in exercising muscles of hypertensive individuals compromising their ability to exercise [64]. This, presumably, leads to persistent afferent activation and hyper-reflexia producing exaggerated pressor responses during exercise but may also contribute to reduced exercise tolerance through fatigue and possibly pain.

There are a wide range of molecules involved in the regulation of blood flow to skeletal muscle during exercise. One major example is ATP. ATP, a potent vasodilator, stimulates the formation of both nitric oxide and prostaglandins [65], which counteract local sympathetic vasoconstriction [66,67]. A further source of nitric oxide production is by mechanically induced signals, including shear stress-activated endothelial nitric oxide synthase [68]. Recent studies have shown that oxidative stress further increases vasoconstriction in skeletal muscle in rats with induced hypertension [69–71], suggesting a reduction/absence of the opposing dilatatory influence of nitric oxide. Taken together, this supports our hypothesis of organ hypoperfusion and blood flow supply–demand mismatch and afferent sensitization from a relatively large vascular bed that can drive neurogenic hypertension (Figure 1).

### • Other contributing afferents & crosstalk

Stimulation of intestinal mechanoreceptors (stretch) or chemoreceptors activates splanchnic nerve afferents resulting in decreases in tissue blood flow in the splanchnic organs (duodenum, jejunum, pancreas, spleen, stomach and liver) and kidneys [72]. This is most likely to be mediated by the sympathetic nervous system since electrical stimulation of intestinal afferents increases sympathetic activity in the splanchnic bed [72]. On the other hand, sympatho-inhibition occurs following the ingestion of a meal. This can augment splanchnic blood flow by approximately 150% above baseline, postprandially [73]. In hypertensive subjects, blood flow to the splanchnic vascular bed is reduced [73], while in obese, hypertensive Sprague–Dawley rats the postprandial sympatho-inhibition and vasodilator effects are abolished. Although this does not separate cause from consequence, it does indicate a connection between splanchnic hypoperfusion, reduced sympatho-inhibition and hypertension.

High blood pressure can induce vascular (arterial/arteriole) hypertrophy [74] as can the increase in sympathetic nervous activity that is associated with hypertension [75–77]. Lumen narrowing, stiffening and exacerbated responses to sympathetic nervous system stimulation may all contribute to elevations in vascular resistance and the hypertensive condition. However, there is also evidence that the arterial remodeling can occur before the development of hypertension as a congenital mechanism in the SHR [78].

Activation of hepatic afferent nerves causes a decrease in blood flow through the hepatic artery again mediated by sympathetic nerves [79]. Activation of spinal cardiac afferents also increases sympathetic activity within minutes [80] and impairs baroreflex control of renal sympathetic nerve activity in rats [80]. Conceptually, this positive feedback can correct hypoperfusion of cardiac tissue thereby reducing the severity of ischemia and decreasing infarct's size in myocardial ischemia injury. As mentioned above, crosstalk between sympathetic and afferent fibers could be a part of a potential mechanism for sensitization, and afferent aberrant discharge as noradrenaline (NA) can activate sensory receptors directly [17], and ATP is coreleased with NA from sympathetic postganglionic efferents [81] and further activates C fibers. Indeed, there is histological evidence of close coupling between efferent and afferent C fibers in human skin [82] and Kopp *et al*. have shown that there is a close apposition of afferent and efferent nerves in the renal pelvis [17]. Finally, activation of the sympathetic supply to the CB causing vasoconstriction [83] could also account for its sensitization and aberrant tone in hypertension.

## The selfish brain hypothesis of hypertension

Cushing's response is enunciated as a physiological nervous system response to increased intracranial pressure that results in Cushing's triad of increased blood pressure, irregular breathing and a reduction of heart rate. Paton *et al*. have proposed that the ‘Cushing mechanism’ is not just a ‘last ditch’ protection for critically ischemic brain, but ‘a physiological mechanism and key regulator of blood pressure’ [84]. This is supported by the observation that in humans high blood pressure levels correlate with increased vertebral artery resistance [84,85]. Thus, could perfusion of the brain be a determinant of the set point of arterial pressure, and is brainstem hypoperfusion a contributing mechanism in neurogenic hypertension? In support of this, increasing cerebral artery vascular resistance activates the sympathetic nervous system in rats [77]. Further, Cates *et al*. have demonstrated that vertebral artery narrowing, increased vertebral artery resistance, a shift to anaerobic metabolism within the brainstem and an exaggerated sympatho-excitatory response to reduced cerebral perfusion, all occur prior the onset of hypertension in spontaneous hypertensive rats [86]. Tissue oxygen levels in the spontaneously hypertensive rat were found to be 15 mmHg lower than in a normotensive rat at the same level of arterial pressure [87]. The transduction mechanism is unknown but could include a central detector of hypoxia, an ‘intracranial baroreceptor’, ion channels sensitive to blood flow or shear stress [86], and may involve ATP and lactate [87]. For example, both traumatic and nontraumatic brain injury can result in paroxysmal sympathetic hyperactivity and hypertension [88].

## Conclusion & future perspective

Although many antihypertensive drugs have a degree of penetrance into the brain, targeting the CNS with drugs causes poorly tolerated side effects and triggers a major clinical challenge of poor patient compliance to medication. According to our hypothesis, we need to consider targets within the PNS and identify those visceral afferents that have become sensitized and tonically activated in hypertension. Establishing the molecular mechanisms for this afferent tonicity would then allow selective drugs (that need not cross the blood–brain barrier) to temper their aberrant discharge. An ideal treatment would be a drug that targets a defined organ with known aberrant activity and blocks this pathological signaling while preserving the physiological operation of the afferent system. For example, selective blockade of purinergic receptor subtype (P2X3) abolished chronic pathological cough but defensive coughing (such as that evoked following aspiration) was preserved [89]. Reducing organ hypoxic-hypoperfusion and improving organ blood flow and oxygen reserve/capacity is another considered approach for treating hypertension. Experimental evidence shows that intrarenal RAS is compartmentalized from the systemic RAS such that intrarenal angiotensin-converting enzyme is not adequately inhibited by plasma concentrations of angiotensin-converting enzyme inhibition in currently used dosages [90]. Given that AngII can cause renal hypoxia and hypertension (see above), RAS inhibition selectively within the kidney may ameliorate hypoxic-hypoperfusion and reduce sympathetic hyperactivity. Furthermore, activation of HIF-1α and HIF-2α has been demonstrated to protect kidneys from hypoxia and progressive injury [91] by making the kidneys less sensitive to hypoxic-hypoperfusion. Notably, the efficacy of HIF activation appears to depend on when it is administered during hypertensive kidney disease [92] stressing the importance of timing relative to disease progression. Taken together, we propose that organ blood flow supply–demand mismatch could be a target for treating the sympathetic hyperactivity in hypertension. The future challenge is to offset arteriolar vasoconstriction, increase organ blood flow and replenish the blood flow and oxygen supply of specific bodily organs.

**EXECUTIVE SUMMARY**

In physiological conditions, blood flow is distributed to all organs in proportion to local oxygen/energy demand.
Blood flow demand can outstrip supply bringing about hypoxic-hypoperfusion especially when vasodilatory mechanisms are compromised.
Aberrant afferent signaling from an organ may occur in response to hypoperfusion or unmet oxygen demand.
Afferent sensitization can lead to excessive reflexly evoked sympathetic vasoconstriction, hypoxic-hypoperfusion and reduced blood flow that leads to further activation of afferent activity.
Recent clinical trials have shown antihypertensive potency of bilateral renal nerve denervation in some drug-resistant hypertensive patients and carotid body afferent denervation in heart failure patients.
Hypertension is associated with an exaggerated exercise pressor response and we propose sensitization and aberrant discharge from skeletal muscle afferents contribute to excessive sympathetic activity.
The brain stem may detect blood flow directly, thereby acting as a CNS determinant of the set point of vasomotor sympathetic tone and contribute to sympathetic excess in the hypertensive condition if it becomes hypoperfused.
We propose that organ blood flow supply–demand mismatch is a major source of visceral afferent drive and can extend across organs contributing to sympathetic excess and hypertension, a process that is exacerbated during nocturnal blood pressure dipping.
